# The Dynamic Changes in the Storage of the Danjiangkou Reservoir and the Influence of the South-North Water Transfer Project

**DOI:** 10.1038/s41598-018-26788-5

**Published:** 2018-06-07

**Authors:** Hai Liu, Jie Yin, Lian Feng

**Affiliations:** 1 Faculty of Resources and Environmental Science, Hubei University, Wuhan, Hubei 430062 China; 2 Southern University of Science and Technology, School of Environmental Science and Engineering, Shenzhen, Guangdong, 518055 China; 3 Wuhan University, School of Remote Sensing and Information, Wuhan, Hubei 430079 China; 4 Jiangxi Provincial Geomatics Center, Nanchang, Jiangxi 330209 China; 5 Guangdong Provincial Key Laboratory of Soil and Groundwater Pollution Control, School of Environmental Science and Engineering, Southern University of Science and Technology, Shenzhen, China

## Abstract

Danjiangkou Reservoir is water source of Middle Route Project of the South-to-North Water Diversion (SNWD) Project, research on the dynamic changes in the water storage within the Danjiangkou Reservoir constitutes an important guide for reservoir water volume management practices. A practical method for estimating the water storage and its dynamics was proposed in this study based on inundated areas from Moderate Resolution Imaging Spectroradiometer (MODIS) observations between 2000 and 2016. The results show that the mean Danjiangkou Reservoir water storage was 10.548 billion m^3^ year^−1^. Significant seasonal changes (*ρ* < 0.01 in *t*-test) were observed with annual minima (~9.610 billion m^3^) occurring between February to July and annual maxima (~11.514 billion m^3^) occurring from August to the following January. Based on the monthly changes in the probability of the water supply, the guaranteed rate of water supply in the second half year (July–December) was higher than that during the first half year (January–June). The water supply in May was greatly deficient. In addition, the full guaranteed rate of the water supply over the past 17 years only accounted for 17.6%. Therefore, reservoir management practices should reduce the water released prior to May and reserve enough water according to the demands of water receiving areas to improve the efficiency of water resource utilization.

## Introduction

Large reservoirs constitute an important part of the surface water cycle and play critical roles in water resource management, flood control endeavors, and climate change at the regional and global scales^1–3^. Large reservoirs may also be involved in interbasin water transfer^4^, and changes in their storage capacities could reflect anthropological effects on the climate, as all of these factors are capable of interacting with and constraining one another.

**Figure 1:**
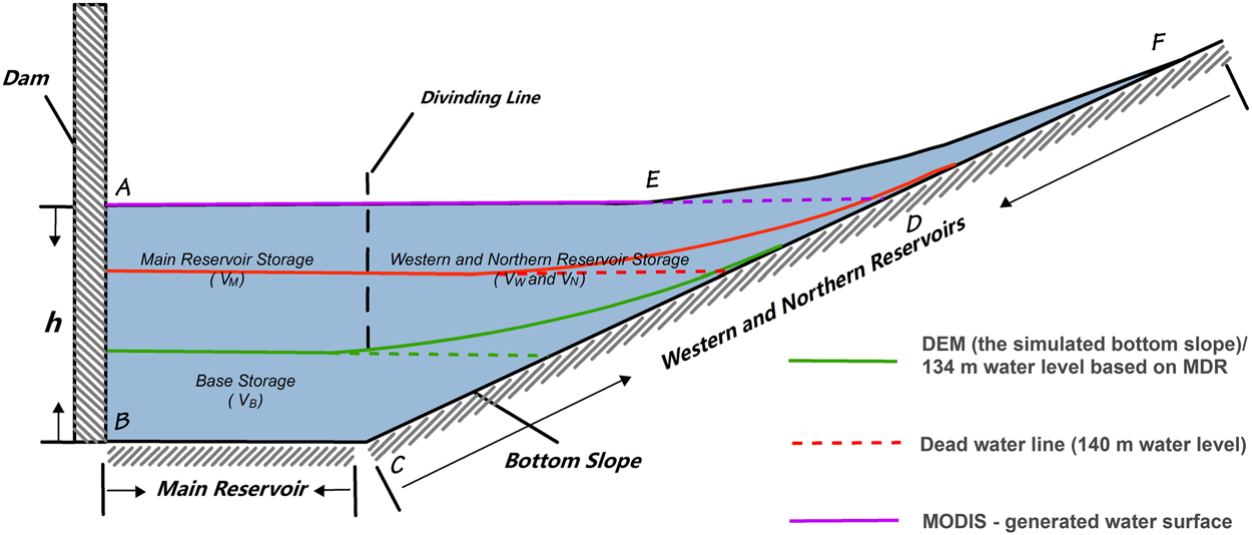
Reservoir storage profile model. The polygon ABCD represent the static water storage; the irregular figure DEF refer to dynamic water storage; the veritable water storage includes the polygon and irregular figure (i.e., the polygon ABCDEF)., According to the terrain condition DEM reflects, this paper divides the Danjiangkou reservoir storage into four parts, including main reservoir storage, western reservoir storage, northern reservoir storage and base storage (V = V_M_ + V_N_ + V_W_ + V_B_).

Reservoir storage, which is the volume between a curved surface and a certain datum, refers to the storage of a reservoir below a certain water level^5^. At present, two types of methods are used to calculate the reservoir storage: (a) When the reservoir storage is considered static, the polygon ABCD in Fig. 1 represents the static storage when the water level is h. This method assumes that the water level is homogeneous throughout the entire reservoir and its estimation is simple and suitable for lake-type reservoirs without an obvious water level gradient. However, it may not be suitable for river-type reservoirs, which may exhibit a significant water level gradient^6^. For example, when the static storage estimation method is used, the water volume within DEF (Fig. 1) will be neglected, thereby causing large errors within the results^7^. (b) The second method considers the water within the closed polygon of ABCDEF (Fig. 1) as the reservoir storage. While this approach is expected to produce a storage estimate with greater accuracy, the calculation process is more complicated^8^, especially the computation for the dynamic reservoir storage between the backwater surface and the horizontal plane at the upstream water level (i.e., the water within DEF). The typical corresponding calculations require hydrodynamic methods that consider various factors, including the upstream water level, the hydraulic index, and the river gradient ratio, and be confronted with challenges^9^.

Due to the influences of climatic conditions, human regulations, and many other factors, both rapid fluctuations and secular evolution in the reservoir water storage are prominent. Therefore, current reservoir storage research is heavily focused on long-term and frequent dynamic monitoring^10,11^. In general, two types of methods are used to obtain dynamic changes in the reservoir storage: (1) traditional gauged water level-based hydrological calculations and (2) spatial information technology-based simulations. The traditional method is closely related to measurements of the water level. The reservoir storage can be obtained by applying an empirical relationship using gauged water level data (namely, the storage curve), and the dynamics of the storage can be monitored using observed changes in the water level. This method is advantageous because the computational process is rather simple and an operational monitoring system is easy to deploy. Unfortunately, reservoir storage curves were typically constructed long ago, and thus, the bathymetry may have changed significantly since their inception, thereby leading to inaccurate storage calculations^12^. Digital photogrammetry can be employed to collect topographic data of reservoir areas during low flows to form a model of the reservoir bottom elevation and calculate the reservoir storage using water level data through an application of the section method^13^. The advantage of this method is that it can obtain reservoir storage results with greater accuracy, which is related to the accuracy of the topography at the bottom of the reservoir. Meanwhile, this method is disadvantageous because acquiring the necessary terrain data requires substantial amounts of manpower and material resources. However, the emergence of spatial information technology, especially the development of remote sensing platforms, makes it possible to collect multiple continuous observations of the Earth’s surface over a broad period of time at low cost. Water bodies exhibit low reflectance in the visible light, near-infrared and mid-infrared bands of optical remote sensing images, simplifying the extraction of relevant water body information^14^. In addition, since water surfaces have been studied by many scholars,several models^15–20^ have been applied to study water changes throughout different regions.

**Figure 2:**
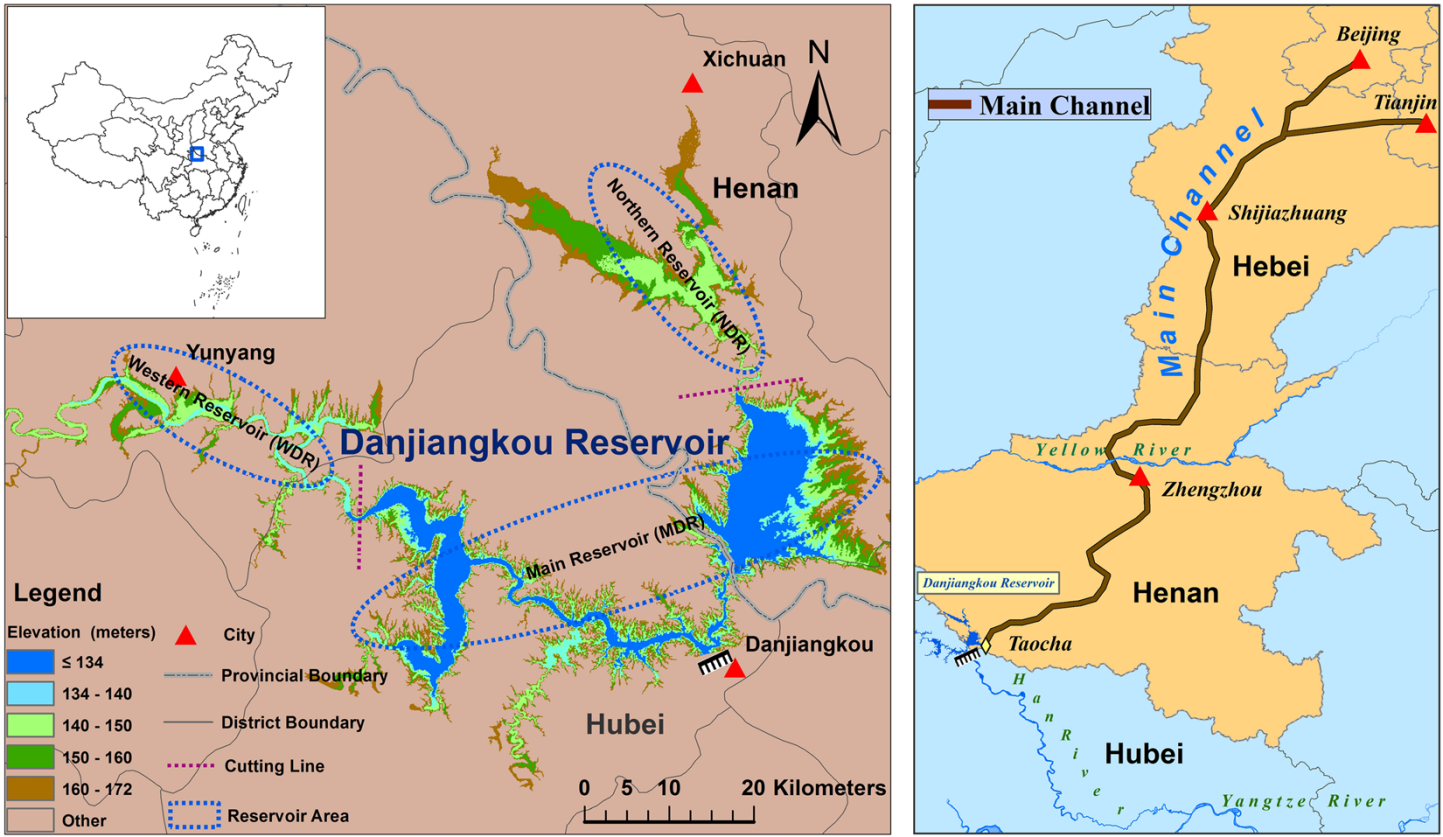
Location map of the Danjiangkou Reservoir and the Middle Route Project of South-North Diversion Project (SNDW). (The map was created using ESRI ArcGIS 10.3, http://www.esri.com/software/arcgis/arcgis-for-desktop).

The Danjiangkou Reservoir (32°36′–33°48′N; 110°59′–111°49′E) is the source of water for the strategic Middle Route Project of the South-North Water Transfer Project^21^ (see location in Fig. 2). The South-North Water Transfer Project is a major interbasin water transfer project in China, and the construction was started in 2003 and the first phase has been finished in 2013, respectively. During the two-year period between 2014 and 2016, the Danjiangkou Reservoir supplied 62.50 billion cubic meters of water for the project, thereby benefiting 40 million people in Beijing and Tianjin and throughout Hebei Province and Henan Province. After the initiation of the first phase of the Middle Route Project, interbasin water transfer within the Danjiangkou Reservoir caused changes in the reservoir storage that could not be ignored. Long-term research on changes in the Danjiangkou Reservoir storage provides a scientific reference for a desirable set of characteristics with regard to reservoir storage changes as well as for the selection of a reasonable water transfer scheme. Ongoing research projects on the water characteristics of the Danjiangkou Reservoir are focused mainly on the water quality and water area in addition to runoff forecasting^22–24^. Few scholars studied dynamic changes in the Danjiangkou Reservoir storage or conducted a comparative analysis of the characteristics both before and after interbasin water transfer; based on former investigations into changes in the reservoir storage and on the characteristics of the Danjiangkou Reservoir^25–30^, they obtained results regarding changes in the Danjiangkou Reservoir storage between February 2000 and December 2016. During the research in this paper, high-temporal-resolution data from the Moderate Resolution Imaging Spectroradiometer (MODIS) sensor onboard Terra were selected to image the dynamic processes of the water surface of the Danjiangkou Reservoir. In addition, a method combining the static reservoir storage with the dynamic reservoir storage was applied to different reservoir sections. Meanwhile, digital elevation model (DEM) data of the reservoir were also utilized. After extracting information from long-term remote sensing images, the changes in the Danjiangkou Reservoir storage and the influences of water transfer on the reservoir storage were analyzed. This research aims to contribute to the optimization of water regulation management for a critical water source in China.

## Data and Methods

### Division of Subreservoirs

The topography of the Danjiangkou Reservoir is complex; the terrain is high in the northwest and low in the southeast^31^. In this paper, the Danjiangkou Reservoir of which is greater than the elevation of the reservoir DEM grid cell was divided into three parts according to the different characteristic of the bottom topography: the main Danjiangkou reservoir (MDR), the western Danjiangkou reservoir (WDR) and the northern Danjiangkou reservoir (NDR). The dividing line between the MDR and the WDR is the Xijun Bridge, and the dividing line between the MDR and the NDR is Shuanglingzhai-Siwan (Fig. 2).

**Figure 3:**
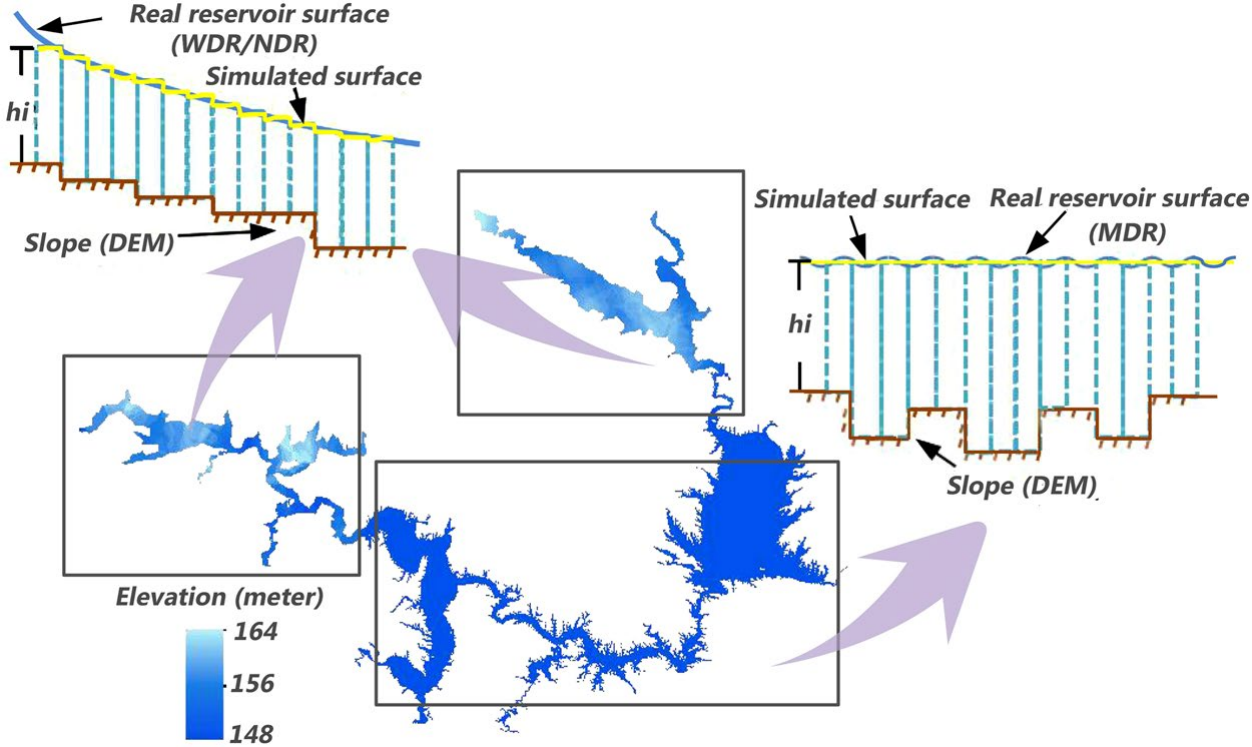
Calculation principle for the storages of the main Danjiangkou Reservoir (MDR), Western Danjiangkou Reservoir (WDR) and Northern Danjiangkou Reservoir (NDR). (The map was created using ESRI ArcGIS 10.3, http://www.esri.com/software/arcgis/arcgis-for-desktop).

### Delineation of Inundation Areas

MODIS 8-day surface reflectance data composites with spatial resolutions of 250 m (MOD09Q1) were used in this study to determine the inundation areas of Dangjiangkou reservoir. A total of 415 composites were downloaded from the NASA Land Processes Distribution Active Archive Center (https://ladsweb.nascom.nasa.gov/), including Terra data from 2000 to 2016.The MODIS 8-day composite products represent the best possible observations during an 8-day period, as they are characterized by high observation coverage, low viewing angle, absence of clouds, etc.

The normalized difference vegetation index (NDVI)^32^ method was applied to extract the water information for the Danjiangkou Reservoir. Generally, when the NDVI is negative, the surface is covered by water, ice or snow. Meanwhile, when the NDVI is equal to 0, the surface is covered by either rock or bare soil, and when the NDVI is positive, the surface is characterized by vegetation^33^. Unfortunately, the threshold of a body of water is usually not fixed, needing to adjust according to actual scope^34,35^. Based on the findings of previous studies and on Landsat images utilized as a reference, the water extraction results were visually examined in this study to select an appropriate threshold. Following visual identification, the threshold value was larger for low flows and smaller for high flows within the reservoir. According to this rule, we again conducted an identification of the water quality from the MODIS data for both high and low flows with a threshold of 0 and a decrease of 0.01. This process was repeated to modify the threshold by comparing with the RGB true color composites.

### Water Storage Estimation Model

Shuttle Radar Topography Mission (SRTM) DEM with a 90-meter resolution are utilized in this study in consideration of the characteristics of the Danjiangkou Reservoir water regime and the data time series. The reservoir storage calculation model used herein quantifies the water surface and the reservoir bottom as an upper subdivision surface and a lower subdivision surface, respectively, based on a given pixel. The upper surface is viewed as the top of the geometry, and the lower surface is viewed as the bottom of the geometry. Both surfaces intersect at the water and land cut-off. The basic idea of the reservoir storage calculation process is to differentiate the entire reservoir into a finite number of regular quadrangular prisms with the same floor space and different lateral edge lengths in a direction perpendicular to the water surface. The length of the lateral edge is the elevation difference between the top and the bottom in the vertical direction. The volume of the regular quadrangular prism is the product of the lateral edge lengths and the floor space. Then, according to the principle of integration, the reservoir storage is equal to the sum of all differential regular quadrangular prism volumes (Fig. 3). The calculation formula for the reservoir storage can be described as follows:

1 $$V={\int }_{S}{H}_{ij}\ast dS$$

where V represents the reservoir storage, S represents the floor space of the regular quadrangular prism, and H_ij_ represents the length of the regular quadrangular prism in the DEM pixel unit (i, j).

The area of the MDR is that of a typical lake-type reservoir, and thus, the static reservoir storage is considered to be the total storage. The principle for calculating the static reservoir storage assumes that there are no water level differences at the reservoir surface, and the observed water level is considered to be the water level of the entire reservoir surface. The calculation model for the MDR can be described as follows:

2 $${V}_{M}=\sum _{i=1}^{m}\sum _{j=1}^{n}({h}_{M}-{h}_{ij})\ast S$$

where *V*_*M*_ is the storage of the MDR, *h*_*M*_ is the mean water level, *h*_*ij*_ is the elevation of the grid at the position of (i, j) on the simulated reservoir bottom DEM, *m* and *n* are the number of pixels in the row and column directions of the DEM, respectively, which participate in the reservoir storage calculation, and *S* is the area of a single pixel (90 m by 90 m).

The WDR and NDR are typical river-type reservoirs. Due to the direction of flow, there is a large average difference (up to 10 meters) between the water levels of the upper and lower parts. Therefore, during the reservoir storage calculation, it is difficult to determine an even water surface to fit the actual surface. To resolve this problem, the dynamic reservoir storage was considered the combined storage of the WDR and NDR. The basic principle for this approach is to find a plane (or a discrete surface of discontinuous elevation change) that fits the surface of the real water representing the top of the geometry in the reservoir storage calculation model. Establishing the equidistant observation points whose elevation value is derived from DEM within the boundary line of the extracted water is feasible scheme to get the elevation of the unbeknown water surface through certain points. After applying Kriging interpolation to the observation points^36^, the simulated elevation surface within the scope of the WDR and NDR was obtained and then combined with the simulated reservoir bottom to form a geometry, the maximum volume of which is the storage of the two reservoirs. The difference between these two calculation models lies in the fact that the top elevation of the water body geometry between the WDR and NDR is not even, whereas the water level in the main reservoir is even. The calculation formula for the storages of the WDR and NDR is as follows:

3 $${V}_{W}=\sum _{i=1}^{m}\sum _{j=1}^{n}(h{W}_{ij}-{h}_{ij})\ast S$$

4 $${V}_{N}=\sum _{i=1}^{m}\sum _{j=1}^{n}(h{N}_{ij}-{h}_{ij})\ast S$$

where V_W_ and V_N_ refer to the storages of the western and northern reservoirs, respectively, h_Wij_ and h_Nij_ refer to the elevations of the picture elements at the position of the Kriging interpolation surface (i, j) of the western and northern reservoirs.

A reservoir is set to maintain a minimum water level (known as the dead water level) to ensure normal operations, including gravity irrigation, smooth navigation, and power generation. According to official data, the dead water level of the Danjiangkou Reservoir is 140 m, and the corresponding storage is 76.50 billion cubic meters^37^. Therefore, the bottom reservoir storage below the 134-meter water level is equal to the difference between the reservoir storage of the dead water level and that between the 134-meter and 140-meter water levels. The corresponding formula is as follows:

5 $${V}_{B}=76.50-V^{\prime} $$

where V_B_ is the bottom reservoir storage, and V′ is the reservoir storage between the 134-meter and 140-meter water levels. The water surface of the 140-meter water level is obtained from the reservoir DEM data, and the reservoir storage for a 134-meter-high water level is 58.90 billion cubic meters according to formulas (1), (2) and (5).

### Probability of the Water Supply Estimation

The water supply guarantee rate generally refers to the probability of occurrence of the year when the expected quantity of the water supply, which is evaluated annually, can be fully met^38^. Due to the variable sensitivity of the South-North Water Transfer Project at different time scales in combination with the reservoir storage calculation based on the MODIS imagery from 2000 to 2016, the concept of the monthly water supply guarantee rate is provided herein to describe the balance state relating the Danjiangkou Reservoir water supply and the monthly water demand under constrained conditions:

6 $${\theta }_{i}=\frac{{S}_{i}}{{D}_{i}}\times 100 \% =\{\begin{array}{cc}0 & {S}_{i} < 0\\ {S}_{i}/{D}_{i} \%  & 0\le {S}_{i}\le {D}_{i}\\ 100 \%  & {S}_{i}\ge {D}_{i}\end{array}$$

7 $${S}_{i}={T}_{i}-{V}_{S}$$

8 $${D}_{i}=\frac{1}{M}\sum _{j=1}^{n}\sum _{k=1}^{m}{W}_{ij}\ast {D}_{k}$$

where *θi* refers to the monthly guarantee rate of the water supply in a month *i*, *S*_*i*_ refers to the water supply in a month *i*, *D*_*j*_ refers to the total water demand of the water receiving area under the restriction of the water supply distribution in a month *i*, *T*_*i*_ refers to the reservoir storage monitored via remote sensing in a month *i*, *V*_*S*_ refers to the reservoir regulating storage, *W*_*ij*_ refers to the total water use of the water receiving area in a month *i* when the water structure type is *j*, *D*_*k*_ refers to the annual water consumption in a region *k*, *M* refers to the annual amount of water transfer under the water transfer scheme, *i* refers to the natural month of water transfer, *j* refers to the type of water used, including life, agriculture, and industry uses as well as supplementary water for ecological environmental usage, and *k* denotes the water receiving areas, which include Beijing, Tianjin, Hebei Province and Henan Province.

## Results

### Analysis of the Relationship between the Water Area and the Water Level of the Reservoir

**Figure 4:**
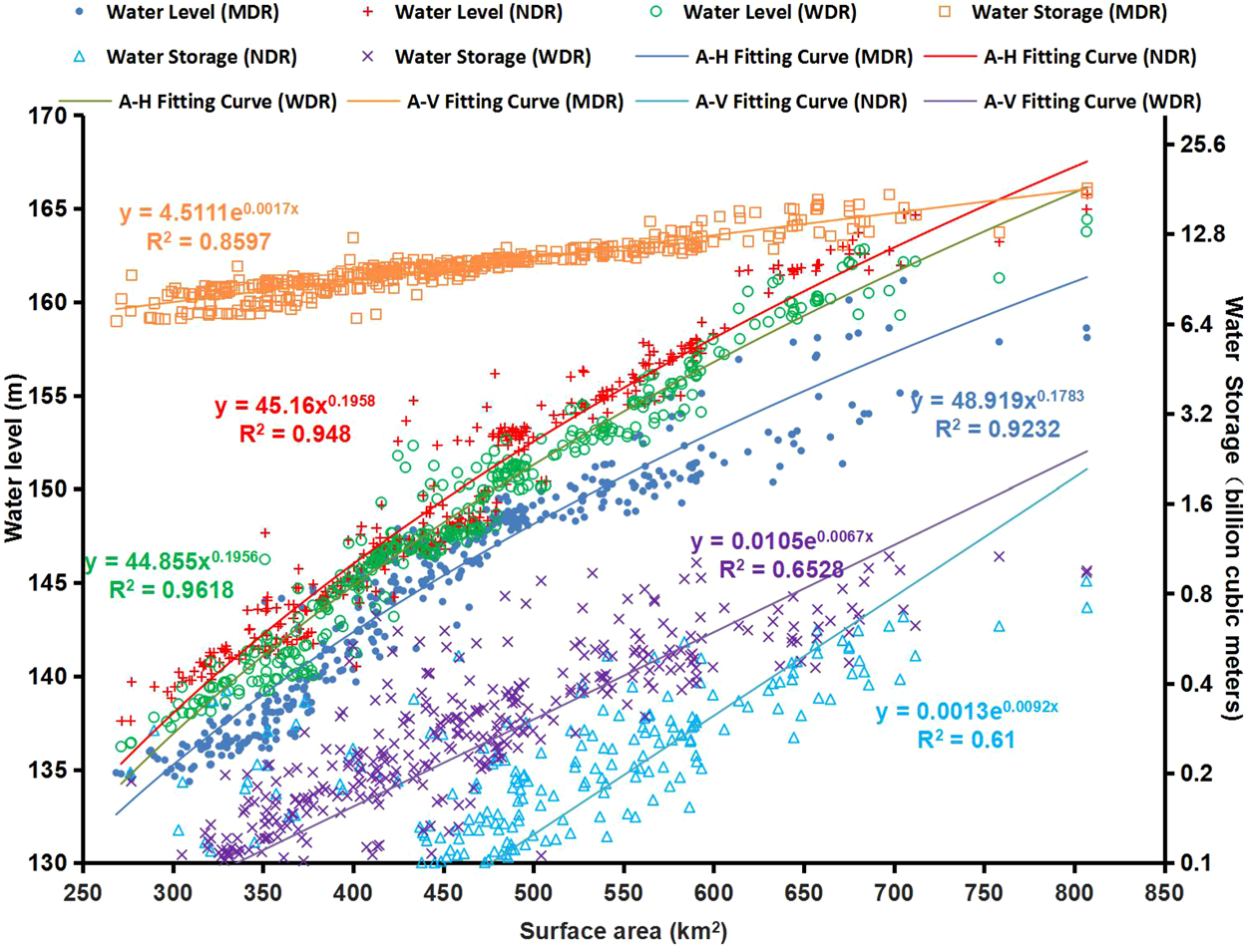
Relationships among the water level, water area and reservoir storage of the Danjiangkou Reservoir. The A-V fitting lines represent the relationships between water area and water storage, and the A-H fitting lines indicate the relationships between water area and water level, respectively.

Time series data for the Danjiangkou Reservoir water area, water level and reservoir storage were monitored via remote sensing from 2000 to 2016. The mean area of the Danjiangkou Reservoir is 452.87 square kilometers; of that total, the mean area of the MDR is 370.37 square kilometers, that of the WDR is 47.16 square kilometers, and that of the NDR is 35.74 square kilometers. The mean water levels of the MDR, WDR and NDR are 145.04 meters, 148.57 meters and 149.81 meters, respectively. The mean storage of the Danjiangkou Reservoir is 10.507 billion cubic meters; of that total, the mean storage of the MDR is 10.030 billion cubic meters, that of the WDR is 301 million cubic meters, and that of the NDR is 175 million cubic meters. In this paper, the water area, mean water level and mean reservoir storage of the corresponding three reservoir areas are used to analyze the change characteristics of each of these three factors (Fig. 4).

As shown in Fig. 4, the mean water level in each reservoir is positively related to the total area of the reservoir, which is concentrated between 300 square kilometers and 600 square kilometers. The mean water level is concentrated between 135 meters and 160 meters. The water level of the MDR is lower than those of the WDR and NDR, while the mean water level of the WDR is lower than that of the NDR. When the total water area is less than 600 square kilometers, the mean water level among the three reservoirs is almost linearly related to the total area and the water level difference between the three reservoirs is small. When the total water area is between 600 square kilometers and 800 square kilometers, the rate of increase in the mean water level among the three reservoirs decreases as the total water area increases and the water level of the MDR is obviously lower than those of the WDR and NDR.

The reservoir storage is also positively correlated with the water area change. From 2000 to 2016, the storage of the MDR was between 6 billion cubic meters and 18 billion cubic meters, accounting for 75.05% of the total storage during the observation period. The corresponding total area of the reservoir was between approximately 35 billion cubic meters and 60 billion cubic meters. In general, the storages of the WDR and the NDR were one order of magnitude smaller than that of the MDR and were mostly under 1 billion cubic meters.

### Changes in Long-Term Time Series of the Reservoir Storage

**Figure 5:**
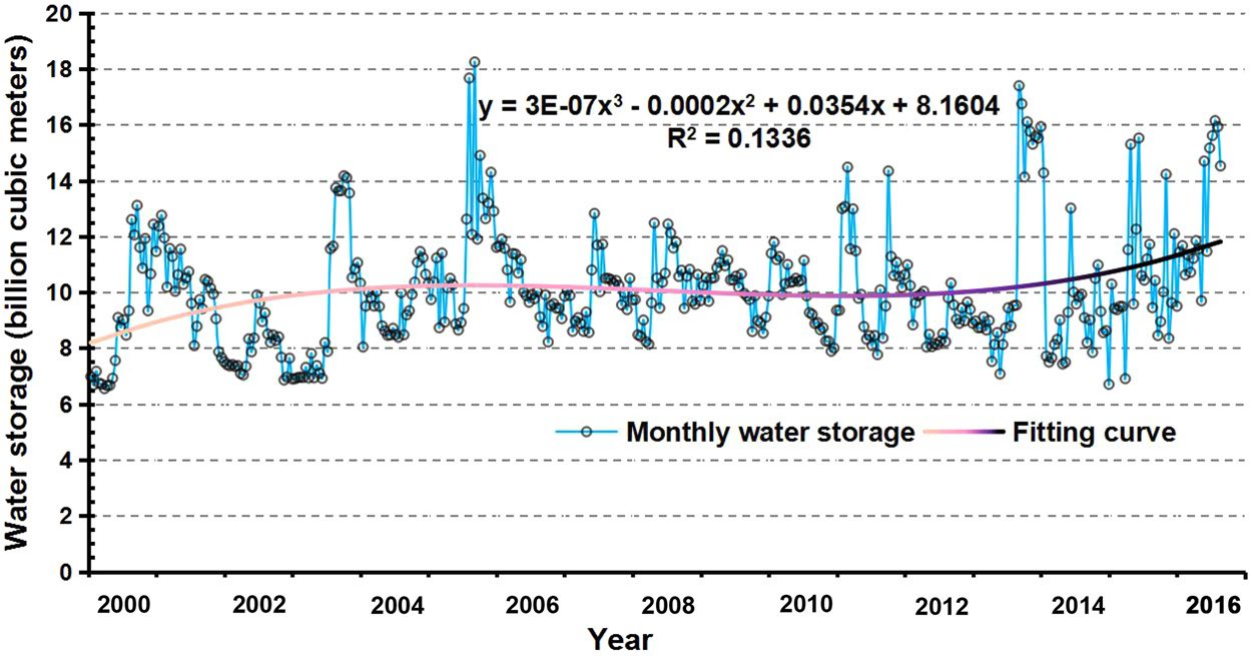
Monthly mean storage of the Danjiangkou Reservoir from 2000 to 2016.

The monthly mean reservoir storage time series, which consists of the Danjiangkou Reservoir remote sensing-monitored storage from 2000 to 2016, is used to analyze the characteristics and trends of the reservoir storage changes (Fig. 5). The Danjiangkou Reservoir storage remained between 6 billion cubic meters and 20 billion cubic meters over the long term and fluctuated by 10 billion cubic meters. However, the standard deviation of the monthly mean reservoir storage *σ* is 2.304 billion cubic meters, accounting for 21.93% of the annual mean storage of the Danjiangkou Reservoir. This result shows that the storage of the Danjiangkou Reservoir exhibits relative stability on an inter-monthly basis. In addition, the storage of the Danjiangkou Reservoir does not fluctuate sharply under climatic conditions and artificial regulations. In addition, the storage of the Danjiangkou Reservoir gradually increased based on the change trend of the monthly mean reservoir storage. The slope of the fitting curve is steeper subsequent to the heightening of the dam, and the trend of the reservoir storage increase is more obvious.

**Figure 6:**
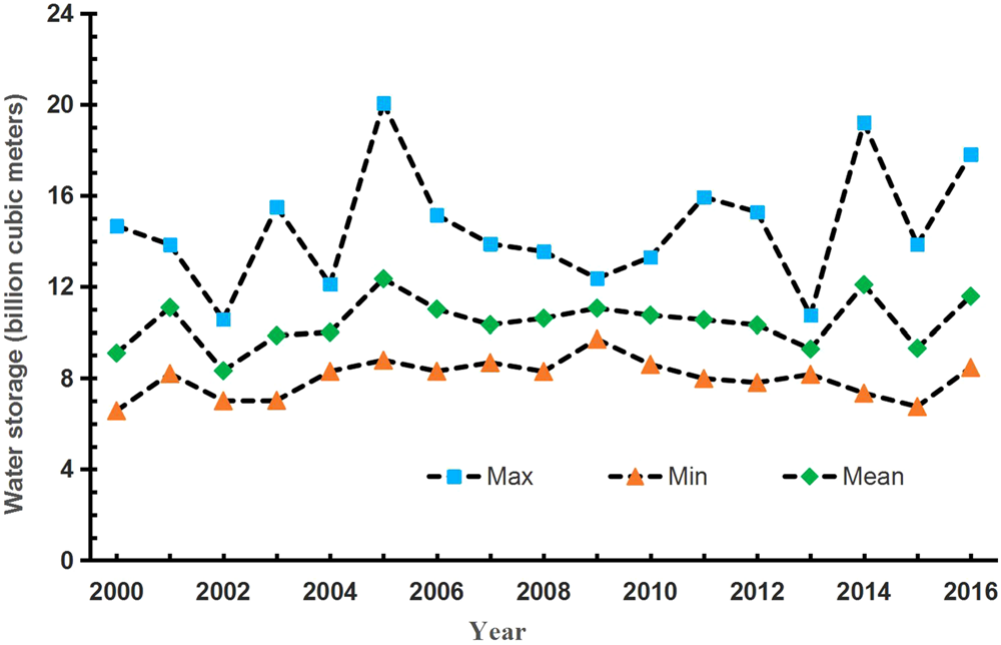
Interannual variations in the storage of the Danjiangkou Reservoir.

Figure 6 shows the maximum, minimum and mean capacities of the Danjiangkou Reservoir from 2000 to 2016. The maximum reservoir storage fluctuated greatly each year, and the maximum/minimum ratio was 1.90. The maximum reservoir storage was ranged between 12 billion cubic meters and 18 billion cubic meters with an average of 14.574 billion cubic meters. The minimum annual reservoir storage in each year fluctuated more slowly, and the maximum/minimum ratio was 1.47. The minimum storage was mainly concentrated between 7 billion cubic meters and 8.5 billion cubic meters with an average of 8 billion cubic meters. The mean reservoir storage fluctuation range for each year was between the maximum value and the minimum value, and the maximum/minimum ratio was 1.48. The mean reservoir storage fluctuated mainly between 10 billion cubic meters and 12 billion cubic meters with an average of 10.447 billion cubic meters. At the same time, the mean annual fluctuation in the reservoir storage is similar to that in the minimum annual reservoir storage for each year, albeit with a greater fluctuation range.

**Figure 7:**
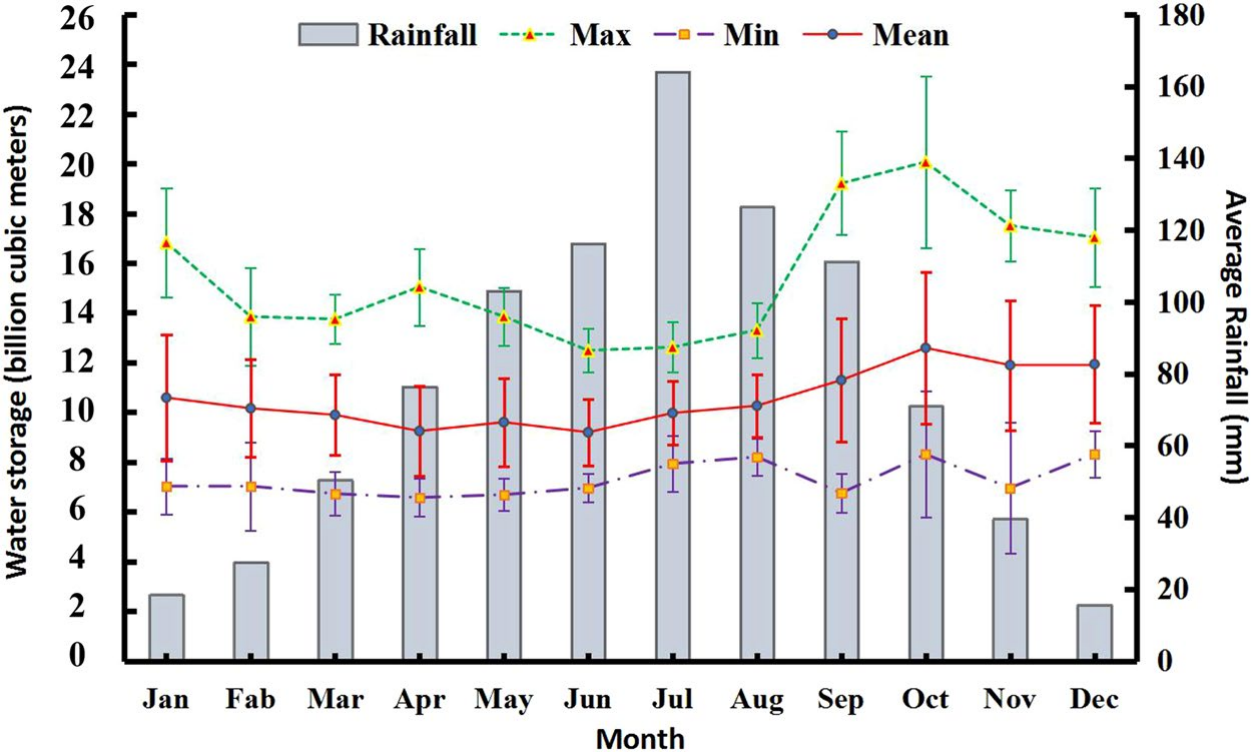
Inter-monthly variations in the storage of the Danjiangkou Reservoir.

Figure 7 shows the changes in the monthly mean storage of the Danjiangkou Reservoir between 2000 and 2016. The fluctuation in the monthly maximum reservoir storage is not significant. The monthly maximum reservoir storage from September to November was more than 16 billion cubic meters, while those in the other months barely exceed 16 billion cubic meters. From the time series of the reservoir storage, the reservoir storage reached 16 billion cubic meters a total of 17 times with the following monthly frequencies: twice in September, 5 times in October, 4 times in November, 4 times in December and twice in January. This finding shows that the monthly maximum reservoir storage was reached more frequently between October and December. The monthly minimum reservoir storage from January to June showed no obvious fluctuations and remained between 6.5 billion cubic meters and 7 billion cubic meters. In addition, the standard deviation *σ* was less than 200 million cubic meters. The fluctuation in the monthly minimum reservoir storage from July to December was significant, and the standard deviation increased by 2.6 times compared with that from January to June. The monthly mean reservoir storage generally decreased first and then increased, while it gradually decreased from January to April. The monthly mean reservoir storage initially increased slightly from April to June, after which it decreased to the minimum reservoir storage for the year. From June to October, the reservoir storage experienced a substantial increase of 848 million cubic meters per month before reaching the maximum reservoir storage of 12.585 billion cubic meters for the year. From October to December, the reservoir storage remained relatively stable. The Pearson correlation coefficient between the monthly maximum reservoir storage and the monthly mean reservoir storage is 0.853 (*p* < 0.05), while that between the monthly minimum reservoir storage and the monthly mean reservoir storage is 0.62 (p < 0.05). The monthly maximum reservoir storage, monthly minimum reservoir storage and monthly mean reservoir storage did not show a significant correlation with the monthly mean rainfall over the observation period; this may have been the result of storage regulations on the Danjiangkou Reservoir, as flood control safety is a priority. The Danjiangkou Reservoir is controlled via pluriennial regulations. Taking July as an example, the rainfall pattern was dominated by long-duration, low-intensity frontal rainfall despite having the largest monthly amount of rainfall of the year, and thus, the reservoir regulation allows for enough time to discharge the water. Therefore, the monthly maximum, minimum and mean reservoir capacities did not increase significantly with an increase in the rainfall.

### Dynamic Changes in the Guaranteed Rate of Water Supply of the Reservoir

**Figure 8:**
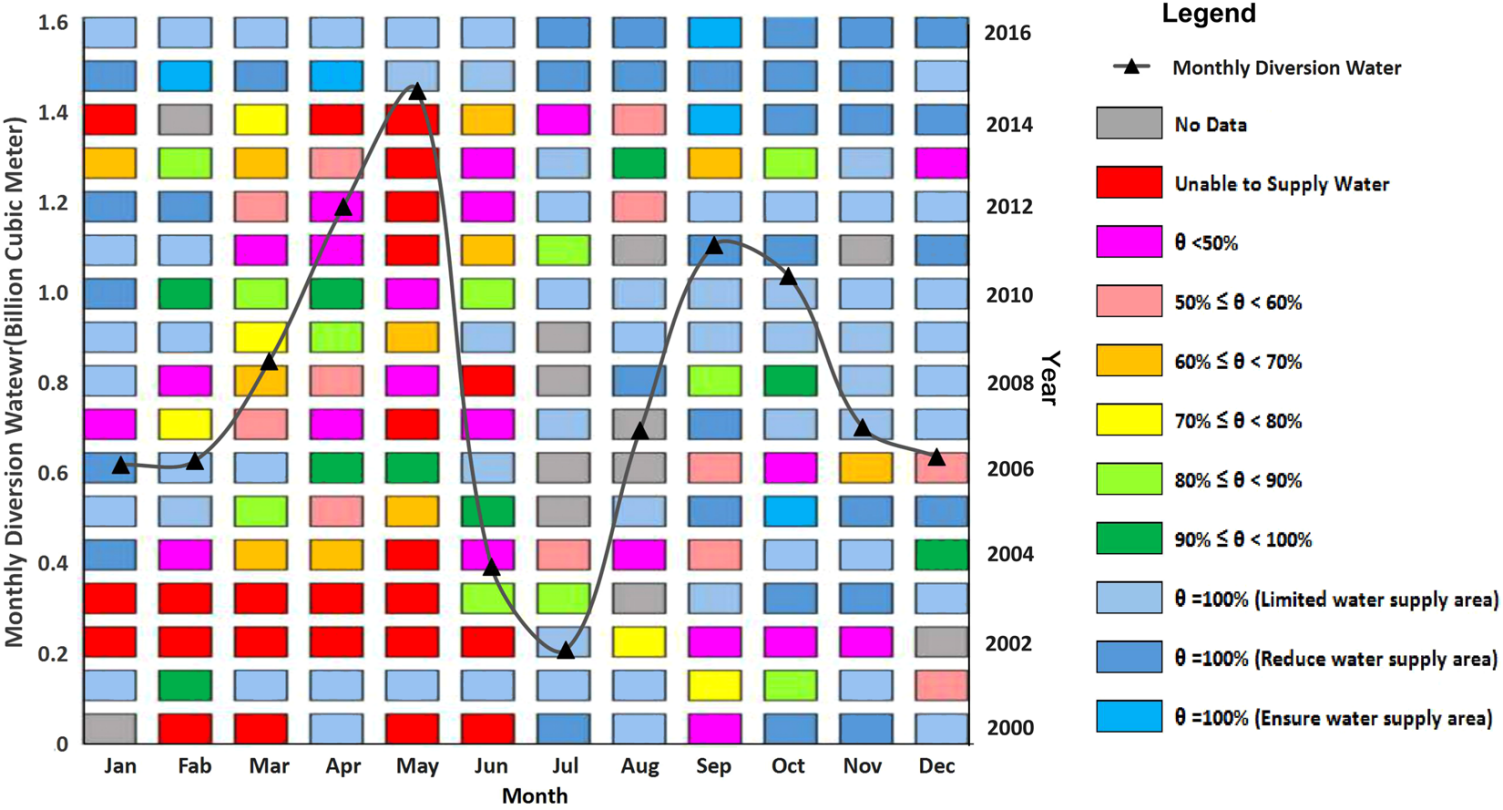
Water supply guarantee rate chart under the assumed conditions from 2000 to 2016.

According to the planning for the Middle Route Project of the South-North Water Transfer Project, the first phase of the project annually transferred 9.5 billion cubic meters of water. The water distribution and regulation are still in the exploratory and experimental stages. The current use of a real-time control strategy only considers the water level and flow of the transport sluice. There is no overall consideration of the storage of the water transfer areas or the demand in the water receiving areas; this lack of consideration is likely to cause an imbalance between the water supply and the water demand^39^. According to the inter-monthly total amount of water used, water use structure and rainfall in the water receiving area^40–45^, the monthly water transfer map of the reservoir was obtained based on the demand of the receiving areas under the constraints of the total annual water transfer in phase 1 and the distribution ratio for the four provinces and cities (Fig. 8).

The inter-monthly water transfer map exhibits a double peak, a valley and a platform. Specifically, the peaks in water usage exist from April to May and from September to October, while the trough in water usage exists from June to July, and a stable platform of water usage exists from November to the following February.

The storage of the reservoir completely met the demands of the receiving water areas in 2005 and 2006 (*θ* = 100%). However, the frequency of *θ* = 100% was slightly lower; most of the time, there was a large gap between the real water level and the flood control level. That is, the 160-meter water level from June 21 to August 20 transitioned to 163.5 meters from August 21 to September 1, 163.5 meters from September 1 to September 30, and then gradually to 170 meters from October 1 onward. The reservoir was able to achieve a better water supply guarantee rate in 2001 and from 2005 to 2010, but it could not completely supply water for more than 3 months in 2002 and 2013 primarily as a result of less rainfall and a related water shortage as well as the unfinished dam heightening project. Overall, the months in which the reservoir could supply water to the water receiving areas accounted for 86.7% of the total, and nearly 50% could supply water completely. When dam heightening project was completed in 2012, the full water supply guarantee rate (61.7%) was higher than that before the dam heightening was finished (39.8%). This result indicates that the Danjiangkou Reservoir can supply water to the north most of the time (although it is possible that it may not always be able to do so) and that it performed better after the dam heightening.

There is a clear distinction between the Danjiangkou Reservoir water supply guarantee rate during the first half of the year (January to June) and that during the second half of the year (July to December) over the 17-year period. The first half of the year contained a period with no water supply. The lack of water was particularly prominent in May when water could not be supplied to the north 41.2% of the time, which is much higher than that during the other months (18%). The water supply guarantee rate was generally higher in the second half of the year, when the highest water guarantee rate was in November (87.5%).

From the steadiness of the water supply, an incomplete water supply was observed 7 times (*θ* < 100%), each of which lasted for more than 6 months, over the 17-year period (excluding missing data): from January to June 2002, January to July 2003, February to September 2004, September 2006 to June 2007, January to June 2013, and March to August 2014. During these periods, it was common that the upstream Hanjiang River Basin experienced less rainfall while the downstream showed a great water demand. For example, from March to August 2014, the rainfall upstream of Danjiangkou was 40% less than that during the same period in many years, even during the flood season, which also sustained high temperatures and little rain. Meanwhile, the water demand for plowing downstream of the Hanjiang River Basin gradually increased. These factors led to the continuous operation at low water levels of the Danjiangkou Reservoir. In addition, Henan Province, which is situated near the reservoir, suffered its most severe drought in 63 years. The ratio of the months with an incomplete water supply that lasted 6 months or more to the number of complete months with an incomplete water supply was 45.7%. This result shows that the Danjiangkou Reservoir may not be able to meet the water needs of water receiving area for a long time once a water shortage occurs.

## Discussion

### Accuracy Analysis and Deficiency

The mean water level and water distribution in the reservoir area were derived from the reservoir areas extracted from the remote sensing images. Therefore, an accurate extraction of the reservoir water area is required for an accurate reservoir calculation. The 250-m MODIS data for the water extraction results were validated using synchronous 30-m Landsat TM/ETM+ images. The results demonstrate that the range and the area of water obtained from the observational data at both high flows and low flows are consistent with each other (the difference in the water area is less than 12.7%) and that the boundary between the land and water coincide well. Due to their higher spatial resolution, the Landsat data can identify features in more detail near the boundary line more effectively than MODIS data. However, the water depths of the mixed pixels along the water boundaries are shallow, and the reservoir storage of such a pixel position is much smaller than the total storage of the Danjiangkou Reservoir, although this does not affect the long-term study of dynamic changes in the reservoir storage.

**Figure 9:**
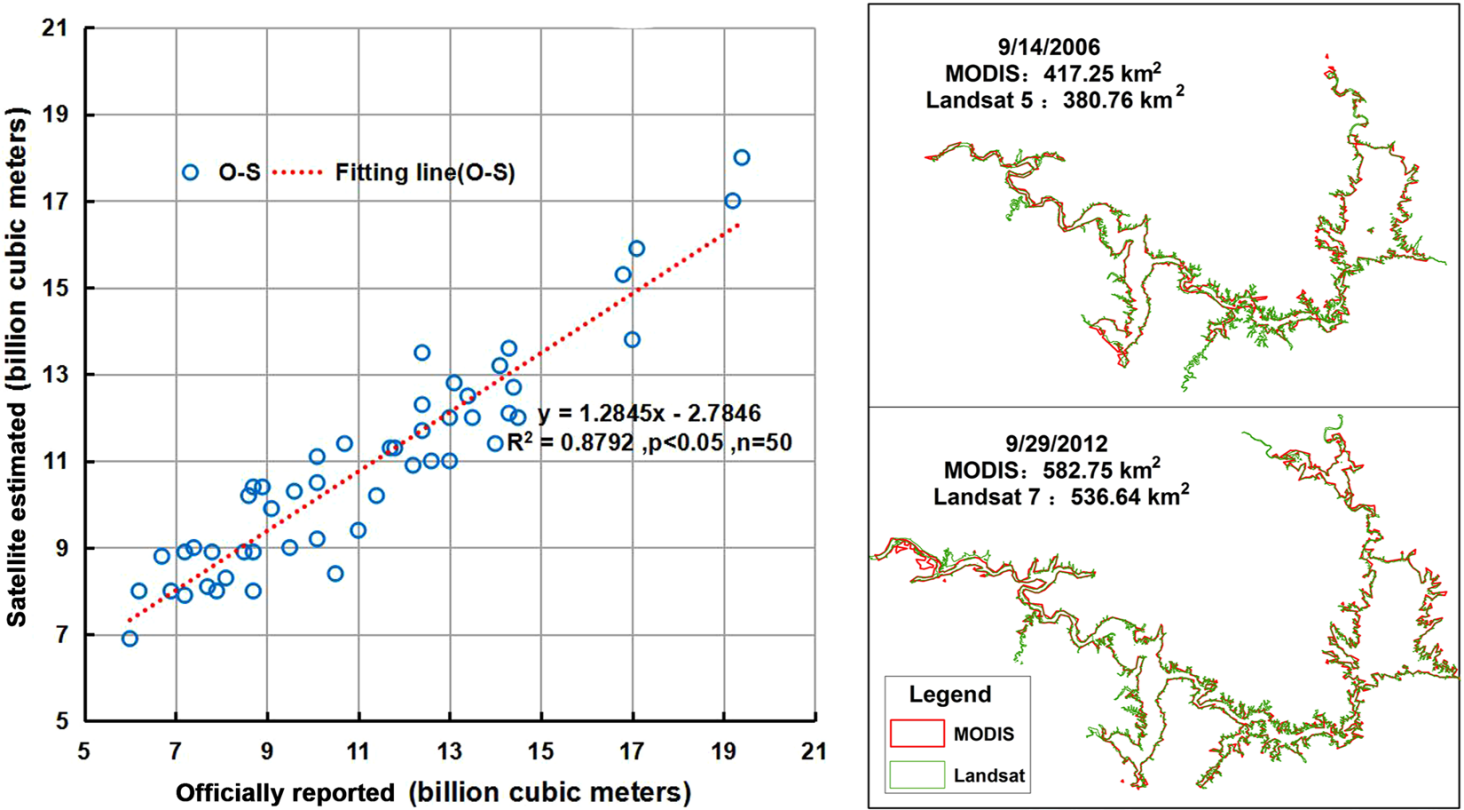
(Left) Scatter plot between satellite estimated and the concurrent officially reported water storage for DanjiangKou reservoir. (Right) Comparison of 250-m MODIS delineated inundation area and that of derived using concurrent 30-m Landsat imagery in two typical high and low water level stages. (The map was created using ESRI ArcGIS 10.3, http://www.esri.com/software/arcgis/arcgis-for-desktop).

Although the MODIS imagery perform well in investigations of long-term dynamic changes in large bodies of water^15,17,20^, their lower spatial resolution can result in the uncertain extraction of boundaries. This occurs because the data resolution can reach up to 250 m only, thereby limiting the image capture and observation of small water bodies such as tributaries flowing into reservoirs and valleys between mountains and influencing the accuracy of reservoir storage calculations. The satellite based water storages agreed very well with the concurrently reported water storage from the official data (Fig. 9). With large range of water storage (6~20 km^3^), with R^2^ reaching to ~0.88, suggesting that the temporal variations in the water storage can be well represented using the remotely sensed estimates.

### Follow-up Recommendations for Danjiangkou Water Transfer

To date, the South-North Water Transfer Project has transferred water stably and safely to the north for two years. In consideration of the reservoir water safety, the water transfer scheme and other factors, the quantity of transferrable water should be improved gradually to achieve the best method of water transfer^46^. At present, the annual mean water transfer has reached 3 billion cubic meters. The Danjiangkou Reservoir is characterized by increasingly frequent fluctuations in its reservoir storage and the presence of a few extreme reservoirs. This finding indicates that the current regulation measures are very likely to have an impact on the storage changes. However, the reservoir storage demonstrates a long-term stability and short-term changes. A slight increase in the mean reservoir storage after water transfer indicates that the reservoir can maintain a certain water transfer potential without crossing the mean reservoir storage threshold over many years.

The Danjiangkou Reservoir has experienced a series of projects and measures to increase its water supply, including dam heightening and storage improvement projects and an optimization of management operations^47^. The water supplies in 2015 and 2016 also illustrate this point, but the historical water storage of the Danjiangkou Reservoir has been affected by interactions between the climate conditions and the results of human activities. The climate conditions in the region are relatively stable, and the human activities are rather continuous; this suggests a clear significance for the historical water supply guarantee rate. The Danjiangkou Reservoir can meet the basic water demands of water receiving areas during the second half of the year, but the deficiency between the water supply and demand is prominent during the first half of the year, especially in May. It is often difficult to intake water from Taocha. This difficulty is related to the guidelines for flood control preceding water transfer, which decreases the reservoir storage to sustain the impacts of the flood season, resulting in a water shortage in May. Therefore, a water transfer plan for the period from March to June should be constructed by analyzing the water use trends of the middle and lower reaches of the Hanjiang River. Under the condition of guaranteeing the water demand for the middle and lower reaches of the Hanjiang River in addition to the water receiving areas, measures should be taken to reasonably reduce the discharged water for a sufficient reservoir storage and to improve the efficiency of water resource utilization.

At the same time, in response to the unsustainable water supply in the Danjiangkou Reservoir, the successful North-South Water Transfer Project of California in the United States can be studied^48^. The “Water Bank” model^49,50^, which is in accordance with the characteristics of the Middle Route Project, can be established accordingly. The principle of this model is to make full use of the wide surface water storage system and the advantages of the aquifer storage space. First, an area into which water can easily penetrate and from which water can be extracted is found, after which underground aquifers are utilized to form large reservoirs. Then, rainfall is injected back into the remaining water supply during the rainy season, the surface water is transferred over long distances, and standard water is transported into the underground aquifer for storage after advanced treatment. Finally, the reservoir water can be used during the dry season or when water is needed^51^.

## Conclusions

In this paper, the long-term dynamic changes in the water storage of the Danjiangkou Reservoir (in the framework of the Middle Route Project of the South-North Water Transfer Project) were studied for the first time. The storage of the Danjiangkou Reservoir from 2000 to 2016 was calculated using Terra/MODIS data and DEM data. From 2000 to 2016, the mean annual storage of the Danjiangkou Reservoir was 10.548 billion cubic meters, the maximum storage was 20.60 billion cubic meters, and the minimum storage was 6.567 billion cubic meters. The water storage of the Danjiangkou Reservoir demonstrated an increasing trend over the observation period. In accordance with the demands of the water receiving areas, the full guaranteed rate of water supply for the Danjiangkou Reservoir was 54.4% from 2000 to 2016 and 100% in both 2015 and 2016. However, there were also periods of water shortage, usually during the first half of the year and especially in May, which highlight a prominent gap between the water supply and demand. Reservoir operation management practices should reasonably reduce the water released prior to May and leave enough water for the demands of water receiving areas.

Water transfer within the Middle Route Project of South-North Water Transfer Project is a multi-function systematic procedure with a complex structure, a high technical difficulty, and a wide range. To date, the water supply to the north of the Danjiangkou Reservoir has reached 3 billion cubic meters, thereby achieving the planned goal of phase 1 of the Middle Route Project. With the assistance of reasonable artificial water transfer, the reservoir storage results show that the Danjiangkou Reservoir is at a safe, stable and reasonable state. However, the recently announced goal of 9 billion cubic meters of transferred water will not be achieved for many years. Therefore, the water quantity, which is another important factor affecting the water transfer safety, should receive close attention.
